# Pharmacogenetics of response to neoadjuvant paclitaxel treatment for locally advanced breast cancer

**DOI:** 10.18632/oncotarget.22461

**Published:** 2017-11-15

**Authors:** Andric C. Perez-Ortiz, Israel Ramírez, Juan C. Cruz-López, Cynthia Villarreal-Garza, Alexandra Luna-Angulo, Esmeralda Lira-Romero, Salvador Jiménez-Chaidez, José Díaz-Chávez, Juan A. Matus-Santos, Laura Sánchez-Chapul, Patricia Mendoza-Lorenzo, Francisco J. Estrada-Mena

**Affiliations:** ^1^ Universidad Panamericana, Escuela de Medicina, Mexico City, Mexico; ^2^ Yale University School of Public Health, Laboratory of Epidemiology and Public Health, New Haven, CT, USA; ^3^ Hospital Regional ISSSTE Puebla and Hospital General Zona Norte SSEP Puebla, Puebla City, Mexico; ^4^ Depto. de Investigacion, Instituto Nacional de Cancerologia, Centro de Cancer de Mama, Tecnologico de Monterrey, Monterrey, Nuevo León, Mexico; ^5^ Instituto Nacional de Rehabilitación, Mexico City, Mexico; ^6^ Unidad de Investigación Biomédica en Cáncer, Instituto de Investigaciones Biomédicas, UNAM/Instituto Nacional de Cancerología, Mexico City, Mexico; ^7^ División Académica de Ciencias Básicas, Unidad Chontalpa, Universidad Juárez Autónoma de Tabasco, Tabasco, Mexico

**Keywords:** pharmacogenetics, paclitaxel, breast cancer, genetic markers, single nucleotide polymorphism

## Abstract

Locally advanced breast cancer (LABC) cases have a varying five-year survival rate, mainly influenced by the tumor response to chemotherapy. Paclitaxel activity (response rate) varies across populations from 21.5% to 84%. There are some reports on genetic traits and paclitaxel; however, there is still considerable residual unexplained variability. In this study, we aimed to test the association between eleven novel markers and tumor response to paclitaxel and to explore if any of them influenced tumor protein expression. We studied a cohort of 140 women with LABC. At baseline, we collected a blood sample (for genotyping), fine needle aspirates (for Western blot), and tumor measurements by imaging. After follow-up, we ascertained the response to paclitaxel monotherapy by comparing the percent change in the pre-, post- tumor measurements after treatment. To allocate exposure, we genotyped eleven SNPs with TaqMan probes on RT-PCR and regressed them to tumor response using linear modeling. In addition, we compared protein expression, between breast tumors and healthy controls, of those genes whose genetic markers were significantly associated with tumor response. After adjusting for multiple clinical covariates, SNPs on the *LPHN2*, *ROBO1, SNTG1*, and *GRIK1* genes were significant independent predictors of poor tumor response (tumor growth) despite paclitaxel treatment. Moreover, proteins encoded by those genes are significantly downregulated in breast tumor samples.

## INTRODUCTION

Breast cancer is the second most common cancer worldwide and the most prevalent cancerous disease among women [1]. Regardless of race and ethnicity, the five-year survival of women with intermediate or locally advanced breast cancer (stages TNM IIB to IIIC) ranges from 50% to 74% [2]. This varying response is common across populations and might be largely influenced by the tumor molecular subtype and the patients’ intrinsic response to chemotherapy [2]. As first-line chemotherapy, taxanes, such as paclitaxel, appear to be more effective than anthracyclines in achieving complete pathological responses, i.e. complete tumor clearance after surgery, (20.9% vs. 12.4% respectively) [3, 4]. Nevertheless, across studies, the range of response rate to taxanes, specifically to paclitaxel, varies significantly from 21.5% to 84% [5, 6]. Part of this variability is explained by genetic traits and might also contribute to the differences observed in the five-year survival [5]. So far studies have only assessed the contribution of some genetic traits on either disease phenotype (i.e. molecular subtypes) or tumor response based on drug metabolism (pharmacokinetics or pharmacodynamics), but there remains a substantial knowledge gap on the variability in response to neoadjuvant chemotherapy, specifically paclitaxel and genetic markers [5].

Paclitaxel has a broad activity spectrum and is used, often in combination, to treat several cancers [5]. It is a microtubule-targeting drug which promotes microtubule stability by binding to the beta subunits of the tubulin leading to the disruption of mitosis and alterations in intracellular communication, resulting in cell death [5]. In breast cancer, paclitaxel is the most common formulation used and researched due to its efficacy but also because of the varying response to treatment and severity of adverse drug reactions after chemotherapy. Several pharmacogenetic studies have assessed these considerable differences in response rates, toxicity, pharmacokinetics, and pharmacodynamics [7]. So far only three major groups of genes or proteins have been researched for breast cancer concerning drug metabolism (*CYP* genes), drug transport (*ABCB1* genes), and site of action (*TUBB* genes) [8]. The evidence suggests that single nucleotide polymorphisms (SNPs) in cytochromes (e.g. *CYP1B1* or *CYP2C8*) and *ABCB1* transporters are associated with better tolerance [9, 10] and reduced efficacy [11] respectively, to paclitaxel. However, most of these genetic markers are non-protein coding or intronic regions, and their relevance as gene regulators is poorly understood [12]. Furthermore, there are no reports of genetic markers on tumor response to paclitaxel in protein coding regions or non-cytochromes non-transporter regions.

Recently Eng *et al.* identified in NCI60 breast cancer cell lines eleven novel single nucleotide polymorphisms on exonic or protein coding regions (in *CFTR*, *ROBO1*, *BTBD12, DCT, SNTG1, SGCD, LPHN2,* and *GRIK1* genes) that are associated with sensitive or resistant phenotype to paclitaxel [13]. Most of them are on novel genes not previously reported for breast cancer. In two of these genes, α1-syntrophin (*SNTA1*, that forms a complex with *SNTG1*) and δ-sarcoglycan (*SGCD*), there is evidence of decreased protein expression in benign breast disease [14, 15]. Taken together, these results support the hypothesis of a differential expression pattern between responders and non-responders at least at the molecular level. However, most of these results are approximations to biological phenomena in bioinformatics analyses performed by the authors. It remains unclear whether these SNPs could have a role in predicting the tumor response to paclitaxel treatment in breast cancer patients. Because of this, we aimed to test if any genetic marker (of those identified by Eng *et al. in silico*) [13] had a significant differential tumor response after paclitaxel among women with locally advanced breast cancer. Moreover, we intended to ascertain if any meaningful changes occurred in protein expression in these genes in breast cancer samples.

## RESULTS

### Cohort characteristics

After follow-up, our cohort had clinical and demographic data on 140 women Table 1. For our inferential analyses, we excluded 29 women since we were not able to ascertain the tumor response to paclitaxel. Thus, our linear models are based on a sample size of 111 individuals. Of the 29, nine subjects did not have a post-treatment mammography. In two cases, the radiologists were not able to produce a quantifiable measurement. The rest were lost to follow-up. For the full sample (*n =* 140), the mean age at diagnosis was 51.3 years (± 10 years), and most of our sample were either overweight or obese. We further stratified the sample by response status (responders vs. non-responders) to test if any significant factor, either demographic or clinical, should be considered in our linear models (See Methods, Statistical Analyses Section). Other than hormonal status and tumor grade, there were no significant differences between responders and non-responders to paclitaxel (Supplementary Table 1). In our cohort, premenopausal women were significantly more likely to be non-responders to paclitaxel ( *p =* 0.017). Also, undifferentiated tumors were significantly more likely to be diagnosed among women who had at least 20% or more tumor growth after paclitaxel monotherapy. Interestingly, in our sample well-differentiated tumors were less likely to occur among women who responded to paclitaxel (Supplementary Table 1). Regarding our genotype data, for all eleven SNPs, the least frequent allele had a minor allele frequency greater than 0.01 (Table 2). Such alleles in our sample are concurrent with already reported single nucleotide polymorphisms for Mexican population in Hap Map [16]. Similarly, to provide evidence of bias in our genotyping, we stratified our results based on response status. All our data follows the Hardy-Weinberg expected distribution (Supplementary Table 2). All our experiments we had a genotyping call rate greater than 95%.

**Table 1:** Description of the sample (*n* = 140)

Characteristic	*N* (%)^*^
Age (years), mean ± SD	51.3 ± 10.0
BMI (kg/m^2^), mean ± SD	28.9 ± 5.1
BMI, *n* (%) (18.5–25) (25–30) (30–35) (35–40) ≥40	34 (24.3) 57 (40.7) 30 (21.4) 16 (11.4) 3 (2.1)
Breastfeeding, *n* (%)	98 (78.4)
Age at first birth (years)^†^, mean ± SD	21.4 ± 5.2
Type 2 diabetes, *n* (%)	19 (13.6)
HTN, *n* (%)	28 (20.0)
Menarche (years), mean ± SD	12.8 ± 1.7
Premenopausal, *n* (%)	66 (47.1)
Metformin use, *n* (%)	12 (10.4)
Hormonal exposure^^^, *n* (%)	32 (25.6)
Pathology report, *n* (%) IDC ILC IDC/ILC Other	122 (87.1) 14 (10.0) 2 (1.4) 2 (1.4)
Neoadjuvant trastuzumab, *n* (%)	19 (13.6)
TNM staging, *n* (%) IIA IIB IIIA IIIB IIIC	6 (4.35) 17 (12.3) 66 (47.8) 38 (27.5) 10 (7.3)
Molecular subtype, *n* (%) Luminal Her2-enriched Triple-negative	84 (68.3) 18 (14.6) 21 (17.1)
Tumor grade, *n* (%) Well-differentiated Undifferentiated Poorly-differentiated	16 (18.6) 35 (40.7) 35 (40.7)

^*^Numbers may not sum to totals due to missing data, and column percentages may not sum to 100% due to rounding.

^^^Being exposed to synthetic estrogen or estrogen-progestin oral contraceptives.

^†^Only patients treated at the National Cancer Institute (*n* = 83).

BMI: Body mass index, HTN: Hypertension.

**Table 2:** Allele frequencies (*n* = 141)

CHR	Gene	SNP	A1^*^	A2	MAF
1	*LPHN2*^*†*^	rs371363	T	C	0.219
3	*ROBO1*	rs997274	C	T	0.112
3	*ROBO1*	rs1355983	G	T	0.146
5	*SGCD*	rs7715464	A	G	0.265
5	*SGCD*	rs931798	A	G	0.250
5	*SGCD*	rs7731517	T	G	0.146
8	*SNTG1*	rs318885	T	G	0.019
13	*DCT*	rs727299	T	C	0.027
16	*BTBD12*^*^*^	rs714181	A	G	0.135
21	*GRIK1*	rs363599	A	G	0.038
21	*GRIK1*	rs457531	T	C	0.101

CHR – Chromosome, SNP – rs ID, A1 – least frequent allele in the sample (exposed cases, see Study design), MAF – minor allele frequency, A2 – most prevalent allele in the sample (unexposed cases, see Study design).

^†^Other aliases: ADGRL2, LPHH1, LEC1.

^^^Other aliases: SLX4.

^*^All least frequent alleles for our sample are single nucleotide polymorphisms for the Mexican population as reported in HapMap.

### Differences between genomic DNA and tumor DNA

To bolster our approach, we also additionally genotyped ten random breast tumor fine needle aspirates to ensure that our results are reproducible in this setting and to confirm that tumor DNA is not varying at these loci. We computed kappa values and percentages in agreement to evidence any genotype mismatch between tumor and peripheral blood DNA. These results are shown in Supplementary Table 3. For all our SNPs, both genomic and tumor DNA are unvarying, i.e. by the same genotyping methodology, we recorded the same genotypes from two distinct tissue samples.

### Unadjusted effects of a set of genetic markers (SNPs) on tumor response to paclitaxel treatment

To test if any genetic marker (those listed in Table 2) had a significant differential tumor response based on genotypes after paclitaxel, we modeled the relative change in tumor diameter as a function of each single nucleotide polymorphism (Supplementary Table 4). All following effects were analyzed taking the ancestral allele as the reference (intercept) and should be interpreted as the unadjusted effect of the alternative or least frequent allele on tumor growth. Assuming a genotypic model of inheritance, genetic markers in the *LPHN2* (also known as *ADGRL2*) and *GRIK1* genes explained much of the variability in tumor response (14% and 10.4% respectively) in our data. The mean effect of at least one C allele in the rs371363 SNP (*LPHN2* gene) is ∼ 25% less tumor burden after paclitaxel (Supplementary Table 4). This effect holds true for the homozygous C/C [–0.248 95% CI (–0.353, –0.144), *p <* 0.0001] and the heterozygous C/T [–0.261 95% CI (–0.433, –0.089), *p =* 0.003]. Interestingly, our data suggests that the T/T genotype is significantly associated with tumor growth, ∼ 66.2% 95% CI (17.1% – 115.4%, *p =* 0.009) compared to the C/C genotype. Regarding *GRIK1*, the rs363599 G/G genotype is significantly associated with ∼35% less tumor burden after paclitaxel monotherapy [95% CI (–0.442, –0.267), *p <* 0.0001]. We observed a similar effect as with the *LPHN2* gene in that the homozygous A/A is significantly associated with increased tumor size despite paclitaxel treatment [1.497 95% CI (0.644, 2.351), *p <* 0.0001]. The rest of our proposed SNPs had a much lesser impact on the variability in the tumor response to monotherapy with paclitaxel and are detailed further in Supplementary Table 4.

### Unadjusted effect of baseline demographics and clinical data on tumor response after chemotherapy with paclitaxel

We also identified a set of clinical and histopathological predictors of tumor response to paclitaxel treatment displayed in Supplementary Table 5. We found a bivariate association with menopausal status, metformin use, tumor histology, neoadjuvant treatment, TNM staging, and molecular subtype. However, these features poorly explained the variability in the response as evidenced by their adjusted R^2^ values Supplementary Table 5. Notably, women who reported ever been exposed to either estrogens or hormonal therapies (estrogen/progestin combinations) significantly responded poorly to paclitaxel treatment compared to non-users. On average, these women had a ∼30% increase in their breast tumor and this effect could range from 11.2% up to 49.6% ( *p =* 0.002). Since this variable explained a greater proportion (compared to the rest in this category) of the variability of tumor response to paclitaxel (R^2^ = 8.2%) and it is known for its role in carcinogenesis, [17–19] we kept this feature in our regression models regardless of its significance.

### Multivariate analysis of tumor response to paclitaxel

**Figure 1:**
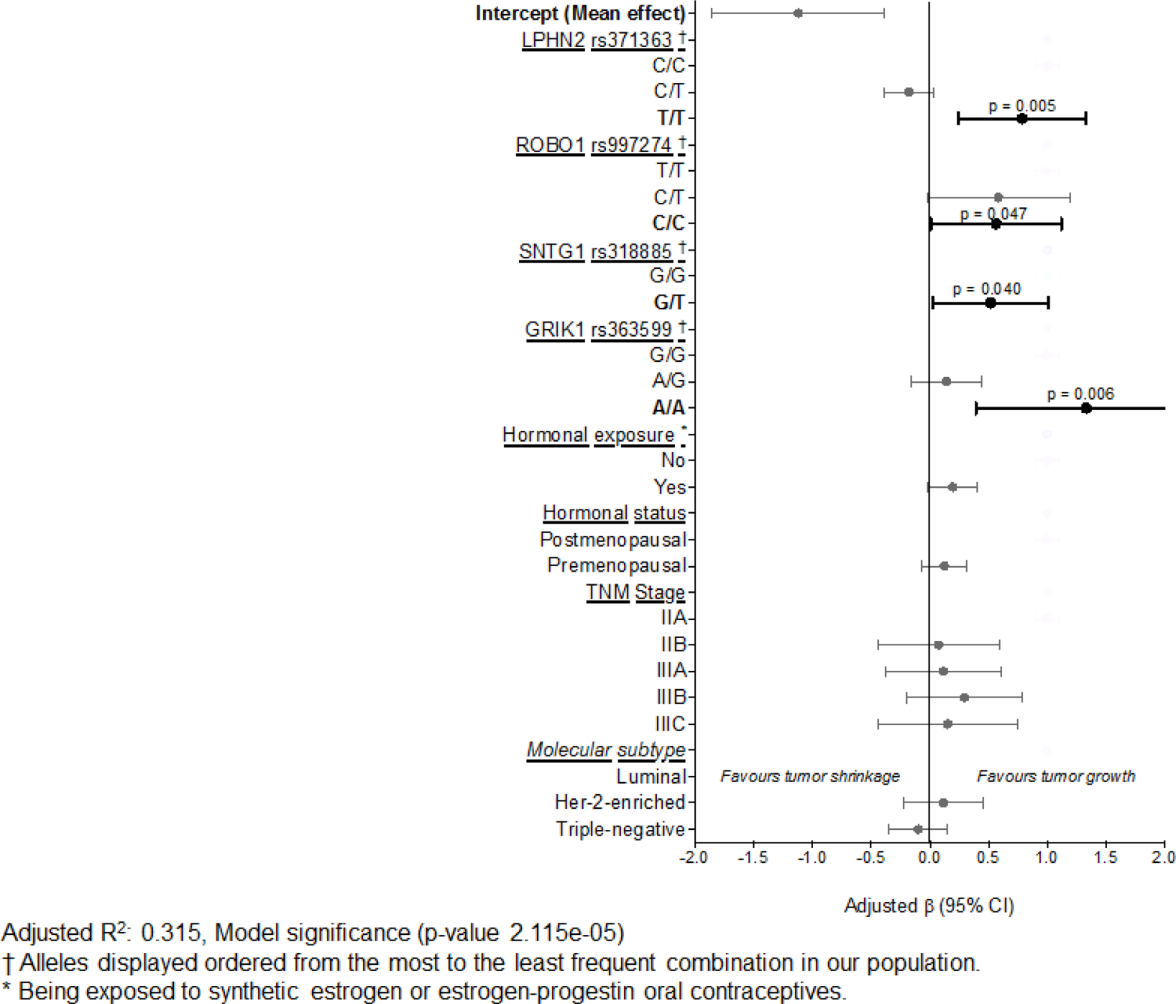
Mean effects of independent predictors of response to paclitaxel treatment

After adjusting for hormonal exposure and status, disease stage, and molecular subtype, in our sample, SNPs on the *ADRGL2* ( *p =* 0.005), *ROBO1* ( *p =* 0.047), *SNTG1* ( *p =* 0.040), and *GRIK1* ( *p =* 0.006) genes are significant independent predictors of tumor response to paclitaxel treatment (Table 3). The mean independent effect of each gene, assuming a genotypic mode of inheritance, is displayed in Table 3, and graphically in Figure 1. Similarly, as explained above in our bivariate models, the following effects for our genetic data were analyzed taking the ancestral allele as the reference and should be interpreted as the independent effect of the least frequent allele on tumor growth despite paclitaxel treatment. Compared to the homozygous ancestral alleles for each gene, the genotype T/T of the rs371363 (*ADRGL2*, also known as *LPHN2*), the C/C alleles of the rs997274 (*ROBO1*), the G/T genotype of the rs318885 (*SNTG1*), and the A/A genotype of the rs363599 (*GRIK1*) are significantly associated with suboptimal tumor response, i.e. tumor growth after paclitaxel treatment (Table 3). Interestingly, the larger effect on poor tumor response is in the *GRIK1* gene (rs363599) as it is positively associated with × 1.33 greater tumor burden despite paclitaxel treatment (95% CI 0.39, 2.227, *p =* 0.047) (Table 3), followed by × 0.78 in *LPHN2* (rs371363) (95% CI 0.24, 1.33, *p =* 0.005). These results are provocative and significantly explain 31.5% of the variability in tumor response (Model significance *p =* 2.12e-0.5).

**Table 3:** Multivariable linear regression model of factors associated with tumor response to paclitaxel (*n* = 81)

Characteristic	Adjusted β (95% CI)	*p*^†^
Intercept	–1,118 (–1.853, –0.383)	**0.003**
LPHN2^*^ [rs371363] CC CT TT	– –0.178 (–0.385, 0.030) **0.786 (0.242, 1.330)**	– 0.092 **0.005**
ROBO1^*^ [rs997274] TT CT CC	– 0.582 (–0.024, 1.188) **0.563 (0.008, 1.118)**	– 0.059 **0.047**
SNTG1^*^ [rs318885] GG GT	– **0.515 (0.025, 1.006)**	– **0.040**
GRIK1^*^ [rs363599] GG AG AA	– 0.141 (–0.161, 0.442) **1.331 (0.393, 2.270)**	– 0.356 **0.006**
Hormonal exposure^ No Yes	– 0.194 (–0.016, 0.403)	– 0.070
Hormonal status Postmenopausal Premenopausal	– 0.122 (–0.067, 0.312)	– 0.202
Stage IIA IIB IIIA IIIB IIIC	– 0.075 (–0.443, 0.592) 0.116 (–0.373, 0.604) 0.293 (–0.196, 0.781) 0.152 (–0.447, 0.750)	– 0.774 0.638 0.236 0.615
Molecular subtype Luminal Her2-enriched Triple-negative	– 0.116 (–0.222, 0.455) –0.100 (–0.353, 0.153)	– 0.496 0.432

Adjusted R2: 0.315, p-value 2.115e-05

– Levels set as reference.

^*^Alleles displayed ordered from the most to the least frequent combination in our population.

^†^p-value for adjusted β significance

^^^Being exposed to synthetic estrogen or estrogen-progestin oral contraceptives.

We took the most common allele for each case and set it as reference. Effects displayed first as those of the intercept for each model.

In bold significant predictors at the 0.05 level.

All these effects follow: $$\Delta \%\text{response}∼\text{SNPs}\left(\begin{array}{l} {AA}_{00}\\ {Aa}_{01}\\ {aa}_{10} \end{array}\right)+\text{Menopausal status}+\text{Stage}+\text{Mol.subtype}+\text{Extposure to horm}.+\varepsilon$$

Additionally, to complement our approach, we ran the adjusted haplotype associations with tumor response for *ROBO1* and *SGCD* (genes with at least two SNPs), but we fail to evidence a significant result after adjusting for multiple covariates (those listed above) (Supplementary Table 5.3 and 5.4). Interestingly, we evidenced a marginally significant result in the *ROBO1* gene (TC haplotype) Supplementary Table 5.1 and 5.2 with a trend that supports a protective effect. However, this haplotype is less frequent in our sample (2.6%) but does warrant future research.

### Protein expression pattern in cancerous breast biopsies of significant genes in the multivariate models

**Figure 2:**
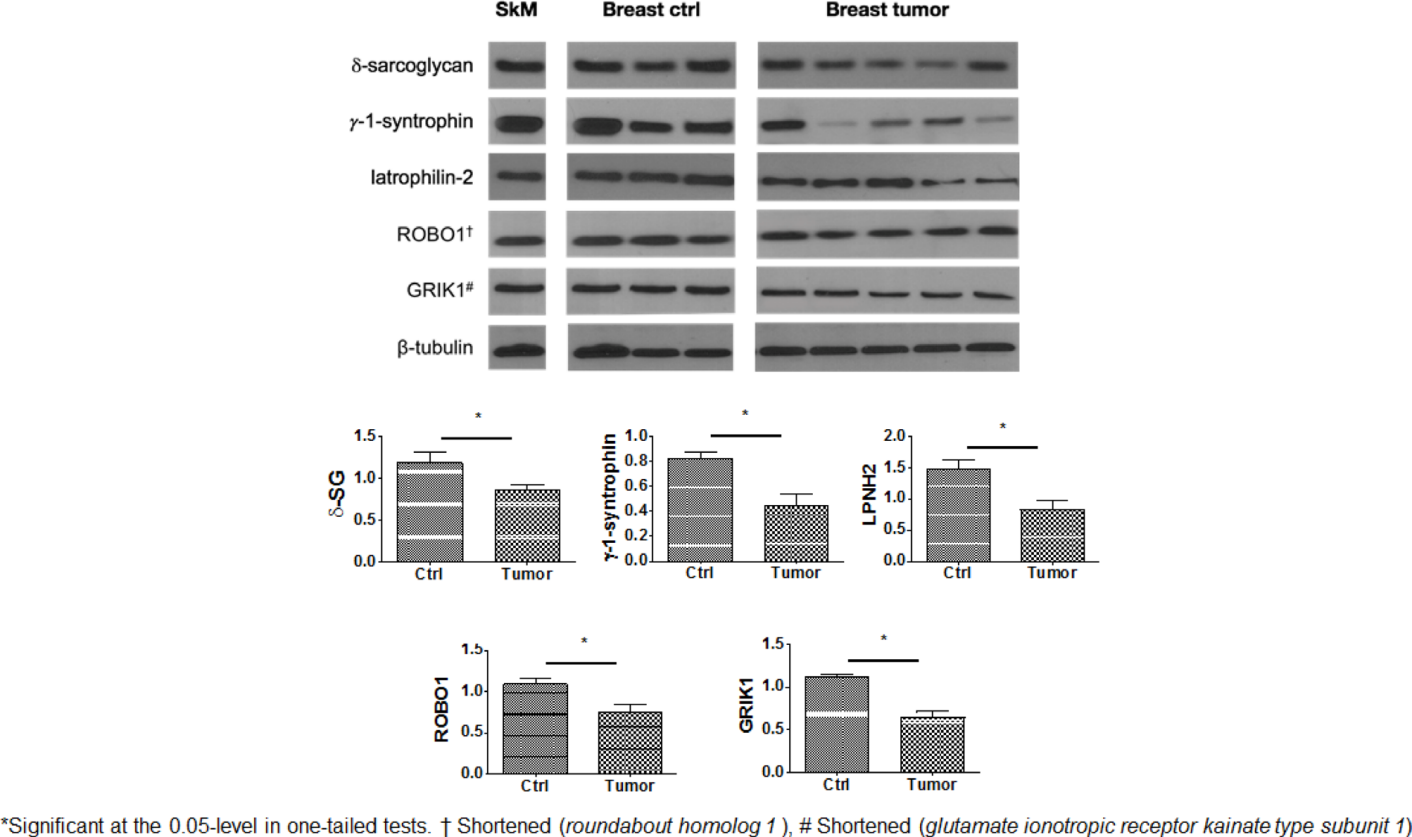
Levels of expression of genes of interest in representative cases of breast cancer (Breast tumor) paired with normal breast tissues (Breast ctrl) Human skeletal muscle (SkM) shown as internal control. All these depictions were run in the same electrophoretic gel but were separated for illustration purposes.

To test if any meaningful changes occurred in protein expression of those genes in our multivariate model (Table 3), regardless of their genotype or tumor response to paclitaxel, we ran Western blots of a series of healthy breast controls compared to a set of breast tumor fine needle aspirates. We additionally included delta-sarcoglycan (gene product of the *SGCD* gene) since it forms a complex with syntrophin-gamma 1 (*SNTG1*) [20], and it has been only reported to be affected in benign breast disease [14]. For all gene products, the median normalized intensities to β-tubulin (AU) in breast tumors appears significantly decreased compared to controls (Supplementary Table 6, Figure 2 in both one-tailed and at the 0.01-level two-tailed tests), i.e. such proteins are downregulated compared to normal breast tissue. Since our results are normalized with beta-tubulin, the difference in median AU units are a proportion and their ratio would represent an estimation of the percent decrease in protein expression between tumor and breast controls. By this interpretation, the most decreased expression in tumor protein relative to healthy tissue is in ROBO1 ∼72.3%, followed by delta sarcoglycan (encoded by *SGCD*) ∼68.9%, and syntrophin gamma 1 (*SNTG1* gene) ∼58% (*SGCD*) (Supplementary Table 6). These results are limited in that we had only results for ten random samples. We suggest a directionality of the differential expression of these proteins in breast tumors, i.e. either up- or down-regulated by non-parametric methods.

## DISCUSSION

The variability in tumor response to paclitaxel monotherapy among women with locally advanced breast cancer is an outgoing issue in oncology research, as the prognosis for such cases is relatively poor, with 5-year survival rates less than 50% (in a systematic review of Hispanic population) despite appropriate treatment [2]. Most of these effects on disease-free survival or mortality are largely influenced by the tumor molecular subtype and the patient’s intrinsic response to first-line neoadjuvant chemotherapy. Genetic variants or markers, such as single nucleotide polymorphisms (SNPs), are emergent intrinsic factors driving this variability in response rate [21]. So far, several studies have used candidate-gene approaches to explore associations with the tumor response to first-line chemotherapy (either taxanes or anthracyclines) [7, 21]. However, these studies have tested the role of genes previously researched in pharmacogenetics studies for other pathologies [22, 8], and do not consider additional markers in non-traditional genes. Thus, there is still an evidence gap in genetic marker research for breast tumor response prediction to paclitaxel treatment.

So far three major groups of genes or proteins have been consistently researched involving paclitaxel’s pharmacokinetics [both metabolism, (e.g. cytochromes), and transport (e.g. ABC transporters)], and pharmacodynamics (site of action e.g. tubulin) [8]. However, breast cancer molecular biology is complex and varied [2] and further exploration of additional markers is warranted. Such non-traditional studies have evidenced two key new significant findings that prone us to pursue our study. First, a novel bioinformatic analysis that uncovered eleven promising SNPs in exonic or protein coding regions (*CFTR, ROBO1, BTBD12, DCT, SNTG1, SGCD, LPHN2,* and *GRIK1* genes) that were previously unknown in breast cancer [13]. Such markers significantly predicted *in silico* a sensitive or resistant phenotype of breast tumor cell cultures to paclitaxel treatment. Second, a new transcriptomic analysis that proposed three novel long-non coding RNAs contributing to paclitaxel resistance (HIF1A-AS2, HOTAIR, and AK124454) [23]. These integrated mRNA-lncRNA signatures effectively classified triple-negative breast cancer (TNBC) patients into groups with low and high risks of disease recurrence [23]. Building upon these works, we sought to test the panel of eleven novel single nucleotide polymorphisms published by Eng *et al.* in women with locally advanced breast cancer. Also, we aimed to predict, using linear modeling, the mean effect or tumor response to paclitaxel based on the patient’s genotype to propose a better allocation of treatment. Preliminary reports on translational medicine and biomarker research in this field are of utmost importance as most patients experience adverse effects to paclitaxel with almost null effects on tumor size or disease progression [5, 7]. We believe our results could contribute to the biomarker research field and might be useful towards building a comprehensive predictive signature of response to paclitaxel, which will facilitate individualized precision medicine.

Moreover, currently, there is no research determining differences in protein expression between responders and non-responders breast tumor samples to neoadjuvant chemotherapy for locally advanced breast cancer, despite a growing public health concern regarding varying response to and toxicity of paclitaxel. Here, we identified five new proteins in breast cancer tumor biology research that could provide useful information on the tumors’ response to chemotherapy. Furthermore, we complemented the findings of Arco *et al.* in that, in our sample, we evidenced a decreased expression of the dystrophin associated protein complex (syntrophin gamma 1 encoded by *SNTG1,* and delta sarcoglycan by *SGCD*) in malignant breast disease compared to healthy tissue. Arco *et al.* previously reported a downregulation of this complex in benign breast disease [14]. We and others have research this protein complex in other tissues, interestingly sarcoglycans (SGs), which consist of α-, β-, γ-, δ-, ε- and ζ-sarcoglycans, are present both in epithelial and in myoepithelial cells of normal breast tissue. In fibrocystic mastopathy and breast fibroadenoma, these proteins are almost absent as evidenced by immunohistochemical and RT-PCR assessments [14]. Syntrophins, also in this complex, and a family consisting of five known homologous protein isoforms (α1, β1, β2, γ1, and γ2) have been mostly reported in brain [24, 25]. Sintrofin-gamma-1 for instance is expressed mainly in neurons bound to other signaling proteins [25]. So far only α1-syntrophin is up-regulated in breast cancer [15]. Here, we report syntrophin gamma 1 (encoded by *SNTG1*) as a new protein down-regulated in breast tumor samples. Further experimentation is warranted to elucidate this complex biological mechanism in breast cancer biology.

One important contribution of our manuscript is our findings on the *LPHN2* gene (also known as *ADGRL2*). In other cancerous diseases such as gastric or colon cancer [26], disruptions in the *LPHN* family might lead to tumor cell resistance to cisplatin. Most of these effects are thought to be epigenetically regulated. However, this gene is a promising biomarker since it’s believed to be in the p53 pathway [26, 27]. We complement the results of Mi-Seong *et al.* in that we show a single nucleotide polymorphism associated with tumor cell activity to a taxane treatment [26]. Secondly, on top of protein expression by western blot, we identified an SNP [rs318885] in the *SNTG1* gene that could contribute to the suboptimal tumor response by impairing breast epithelial cell adhesion, thus facilitating a malignant transformation, [14] or by decreasing the stability of the mitotic microtubules leading to reduced paclitaxel binding and overall effect [28]. In support of these hypotheses, is the expression of the dystrophin associated protein complex, which includes *SNTG1* and *SGCD* (whose SNP was not statistically significant, but we evidenced a decreased protein expression), in breast cells. Specifically, β-sarcoglycan, also part of this complex, colocalizes with α-tubulin (site of action of paclitaxel) [29]. These results open a field that merit in–depth experimentation, especially since the *SNTG1* SNP is located in an intronic region associated with several transcriptional factors, most of them involved in the transcriptional misregulation of cancer [30].

We also contribute to *ROBO1* findings on breast cancer research and on tumor biology. This gene, along *Slit2*, has a well-established role in breast development and morphology [31]. A down-regulation or loss of both is associated with hyperplastic changes in epithelial cells and desmoplastic alterations in the surrounding stroma [32]. Moreover, this decreased expression correlates with poor overall survival and disease-free survival. Previous observations suggest that *ROBO1* can serve as a prognostic biomarker of breast cancer and brain metastasis [31]. Here, we observed one SNP [rs997274] predictive of the tumor response to paclitaxel in our sample. It is of special interest since this SNP has been shown to modulate the transcriptional activity of nuclear hormone receptors through FoxL1 [30]. This factor is involved in *ESR1*-mediated transcription (required for *ESR1* binding to the *NKX2-1* promoter in breast cancer cells), of the *RPRM* promoter, needed for estrogen-induced repression in these cells. It is known that the *RPRM* regulates apoptosis by inhibiting the expression of *BCL2* and regulates cell cycle by activating expression of *CDKN1B*, alone or in conjunction with *BRCA1*. Taken all together, these findings might be indicative of additional pathways to the classic PI3K/Akt/β-catenin/MMP-9 signaling that are contributing to the sensitive or resistant behavior of breast cancer cells to paclitaxel [31]. *ROBO1* is a strong candidate towards becoming a prognostic biomarker supported by our findings and those of Eng *et al.* that described differences in mRNA expression between sensible or resistant NCI60 cancer cell lines [13]. Finally, we present interesting results on the *GRIK1* gene. The glutamate receptor, kainite 1 protein (encoded by *GRIK1*), is involved in the glutamate signaling. In a previous study, an intronic SNP was identified as a susceptibility variant in hepatitis B virus (HBV)-related hepatocellular carcinoma development [33]. Moreover, it has been proven that the inhibition of glutamate release and/or glutamate receptor activity can inhibit the proliferation and/or invasion of tumor cells in triple-negative breast cancer [34]. This is the first report to have found a SNP of *GRIK1* [rs363599] as a major independent suboptimal factor for tumor response to paclitaxel treatment. These findings support the importance of the glutamate signaling pathway in cancer development.

There are numerous strengths as well as certain constraints inherent to our study. Among our main strengths, is the framework we have established. Since paclitaxel is used for other cancerous diseases such as advanced ovary carcinoma, [35] non-small cell lung cancer, [36] and even AIDS-related Kaposi sarcoma, [37] in those settings our results could potentially be extrapolated and replicated. Moreover, we have considered not only bivariate and adjusted associations between genotypes (SNPs) and tumor response to paclitaxel treatment (phenotype) but have also evidenced a marginal association in haplotype analyses. These results warrant future research and replication in other populations. However, we were limited in the number of cases we analyzed in our cohort. We are confident that our results are promising, though preliminary. All our analytical approach and sample size calculations were done to test any significant change in tumor measurements greater than 40%. For all our results, we had optimal statistical power to detect these differences; therefore, we do believe these results merit further validation in other populations, especially to test if any of the markers have an effect that we were underpowered to detect. Also, we excluded women with inflammatory breast disease, since it was not possible to produce a quantifiable measurement for our linear modeling. Future studies could take our results and apply a different analytic strategy to test if any of these SNPs could have a role in these cases. Also, additional considerations in study design are warranted, for instance, survival analyses could be implemented to further validate this SNP as prognostic markers. Moreover, for our Western blot data, we suggest five new proteins that might be decreased in breast tumors compared to normal tissue. We analyzed a small sample using nonparametric methods. Our results given this constraint might be indicative of a directionality of the effect in a larger study. Regardless, our results are promising and warrant future research.

## METHODS

### Study design and population

We performed a cohort of 160 Mexican women aged 18 and older treated for measurable breast cancer (Stages IIA – IIIC) at the National Cancer Institute in Mexico City (Site A) and a tertiary referral hospital in the city of Puebla (Site B) recruited over a period of two years from 2013 to 2015. Our inclusion criteria were:

Women aged 18 years old or older.

Patient naïve to any chemotherapy.

Breast cancer clinical stage IIA – IIIC.

Breast tumor with a diameter greater than 2 cm.

Patientsʼ wish of a conservative breast surgery.

Candidate for neoadjuvant chemotherapy based on paclitaxel.

Available clinical and imaging in the electronic medical records.

Informed consent for the full cohort and genetic testing.

At baseline, we collected demographic and clinical features as well as imaging, and histology data from electronic medical records. All patients were naive to any chemotherapy and agreed to participate after informed consent. We obtained IRB approval following the Declaration of Helsinki from both clinical sites and all patient data was handled as directed by the HIPAA. We started the follow up from the first cycle of paclitaxel and continue onwards after four cycles or until the patient was lost to follow-up during that time. All women received a conventional chemotherapy scheme starting with paclitaxel. The dose and schedule of paclitaxel were 80 mg/m^2^ given in 12 weekly doses [5]. We excluded cases with peripheral neuropathy or those having received other chemotherapy regimen added to paclitaxel. For each eligible case to determine the response to treatment, we performed a digital mammogram/ultrasonogram and gamma gram at the start of the follow-up and after having completed four full cycles with paclitaxel as monotherapy. The patients then resumed the standard care as directed by international guidelines [3]. To allocate exposure, we collected a blood sample for genotyping and requested the pathology department a sample of the fine-needle aspirate that was taken at baseline for Western blot. All samples were kept under optimal conditions (blood at –20C, tissue samples at –70C) at Site A. Exposed cases, in the cohort, were those with the minor allele genotypes of a panel of eleven single nucleotide polymorphisms genotyped at baseline listed in Table 2 (Allele frequencies) (See Allelic discrimination assays). These set of SNPs, known to be related to tumor cell sensitivity to paclitaxel, were chosen from a published work by Eng *et al.* done *in silico* using data from cell cultures [13]. Our internal comparison group were those women who were carriers of the ancestral (or common variant) allele for the Mexican population (Table 2, Allele frequencies). All cases were recruited from the breast cancer clinic in both sites during their first visit (*n =* 140). We are not able to determine the reason for non-participation – patients could have dropped out of the study, be lost to follow-up, or did not consent. However, there were no notable differences between participants and non-participants, regarding demographics, clinical site, and baseline breast cancer stage. Women who participated in the protocol were from the central region of Mexico. We did not have any cases from northern states, which have a much higher incidence of breast cancer compared to southern states [38]. Given our completeness in baseline demographics and clinical data from electronic medical records, our descriptive analyses are based in 140 cases. After our exclusion criteria, we eliminated 29 from our inferential analyses. Thus, our linear regression results are based on a sample of 111 individuals.

### Allelic discrimination assays

To allocate exposure status in our cohort, we extracted genomic DNA (gDNA) from peripheral blood samples taken at the start of the follow-up. We then immediately processed them using the FavorPrep™ Blood/Cultured Cell GENOMIC DNA Extraction (FAVORGEN^®^) kit following the manufacturers’ specifications. DNA concentrations were quantified using the Spectrophotometer Multiskan GO (Thermo Fisher Scientific Inc., Wilmington, DE, USA). For each case in the cohort, we performed TaqMan^®^ assays (Applied Biosystems, Foster City, CA, USA) targeted to eleven single nucleotide polymorphisms previously published by Eng [13]. To ensure reproducibility and precision of our data; all assays were done in duplicate by blinded experienced laboratory technicians (ALA, ELR). We allocated a genotype to each case following conventional methods of melting curve analyses for real-time PCR with the PikoReal™ Real-Time PCR System (Thermo Fisher Scientific Inc., Wilmington, DE, USA).

### Tumor DNA extraction

To bolster our approach, we additionally extracted gDNA from ten random breast tumor fine needle aspirates taken at baseline using AllPrep^**®**^ DNA/RNA/Protein Mini Kit (QIAGEN Inc, Germantown, MD, USA) of cases in our cohort following the manufacturers’ specifications. We then performed all allelic discrimination assays as detailed above.

### Western blot

To address if any genetic marker [those significant SNPs in our multivariate model, (See below)] influenced protein expression, regardless of their genotype or tumor response to chemotherapy, we extracted proteins of five breast cancer specimens. We included two controls for our Western blot data. First, a human muscle biopsy from a cadaveric donor, as a positive or internal control where all those proteins are known to be abundantly expressed. Second, a healthy breast control to which compare the tumor data. Our controls were those women suspected of a benign breast disease (either fibrocystic mastopathy or breast fibroadenoma) in whom the oncologist or oncology resident performed a breast biopsy. A-posteriori the breast pathology department confirmed in these cases the findings not to be either benign or malignant breast disease but normal breast tissue. Out of these tissues, approximately 10 mg of tissue was homogenized in RIPA buffer (Thermo Fisher Scientific Inc., Wilmington, DE, USA) supplemented with proteases inhibitors (Sigma-Aldrich) using a polytron. Equal amounts of protein (20 μg) were electrophoretically separated in 10–14% gradient SDS-PAGE and then electrotransferred to PVDF membranes using a semi-dry immunoblotting system (Bio-Rad). All membranes were blocked for nonspecific binding for 60 min with 5% non-fat milk in T-TBS. The PVDF membranes were then incubated overnight at 4°C with anti-delta sarcoglycan (GeneTex GTX53783), anti-syntrophin gamma 1 (GeneTex GTX10079), anti-LPHN2 (Abcam, ab209548), anti-Robo1 (GeneTex, GTX114103), and anti-GRIK1 (Abcam, ab118891) antibodies. Then, the PVDF membranes were 3X washed with T-TBS and conjugated with an HRP specific secondary antibodies (Cell Signaling Technologies) and developed using the chemiluminescence ECL kit from Amersham. All Western blots were normalized with a loading control (anti-β-tubulin), and compared amongst themselves and with a positive control (human skeletal muscle lysate) (Cell Signaling Technologies). Densitometric values of the digitalized gels were acquired using the ImageJ software [39].

### Unit of analysis

Our outcome of interest was the relative change in tumor size pre- and post-paclitaxel calculated as the percent change in tumor or $\Delta \%\text{response}=\frac{\left({\text{Phase}\;2}_{\text{Postpaclitaxel}}-{\text{Phase}\;1}_{\text{Prepaclitaxel}}\right)}{\left({\text{Phase}\;1}_{\text{Prepaclitaxel}}\right)}.$ Where: Phase 1 is tumor greater diameter at baseline, and Phase 2 after four cycles paclitaxel. All these measurements were assessed by experienced radiologists from the Mastology department at the National Cancer Institute in Mexico City.

### Statistical analyses

We began by describing demographic characteristics and tumor features. Next, we assessed significant differences across between respondents and non-respondents to paclitaxel. For the sole purpose of Supplementary Table 1, we defined non-responders as those women who had at least had a 20% increase in their breast tumor. This measure is similar to what has been used previously in the literature [40]. Next, following the approach taken by Bastien [41], we explored the unadjusted associations of critical clinical and demographic characteristics with the continuous response to paclitaxel treatment (Δ*% response*) using linear modeling. These tests followed:$\Delta \%\text{response}=\frac{\left(\mathrm{Post.taxol}-\mathrm{Pre.taxol}\right)}{\mathrm{Pre.taxol}}∼\text{Clinical}/\text{Demographic features}+\varepsilon .$

We then included those significant predictors at the 0.1-level or those variables with clinical relevance in a multivariate linear regression model.

For our genotype data, we began by describing allelic and minor allele frequencies. We then used PLINK Version 1.9 [42] to examine which type of inheritance to assume in our linear models. We choose that pattern of inheritance which showed significant association with the continuous change in tumor size after paclitaxel (Data not shown). Similarly, we first explored the unadjusted association with Δ% *response* using linear models who followed:

$$\Delta \%\text{response}=\frac{\left(\mathrm{Post.taxol}-\mathrm{Pre.taxol}\right)}{\mathrm{Pre.taxol}}∼\text{SNP}\left(\begin{array}{l} {AA}_{00}\\ {Aa}_{01}\\ {aa}_{10} \end{array}\right)+\varepsilon .$$

Where: AA is the most frequent allele in our population, taken as reference. We then selected those genotypes with a significant association at the 0.05-level and included them in a final multivariate linear regression model. All these models followed standard linear regression assumptions (Data not shown) and were computed in SAS Version. 9.4 [43]. To ascertain, if our genotype results from peripheral blood samples were different from tumor DNA at each SNP locus, we calculated kappa values and percentages in agreement. Kappa value*s >* 0.8 would, therefore, indicate almost perfect agreement between tests in different tissues, i.e. having identical genotypes in both experiments. Finally, we described our Western blot data with a two-sample median score test, to compare the normalized intensities to β-tubulin (AU – arbitrary units) across benign and malignant breast tumors. All our results are presented and detailed following the REMARK guidelines for reporting tumor biomarkers [44].

## SUPPLEMENTARY MATERIALS TABLES



## Abbreviations

LABC: Locally advanced breast cancer
SNPs: Single nucleotide polymorphisms
95%CI: Computed 95% confidence intervals
AU: Arbitrary units (Normalized intensities to β-tubulin)
gDNA: Genomic DNA
RT-PCR: Reverse Transcription Polymerase Chain Reaction
SGs: Sarcoglycans
*SGCD*: gene encoding for delta-sarcoglycan
*SNTG1*: gene encoding for gamma-syntrophin 1
*LPHN2*: gene encoding for latrophilin-2
*ROBO1*: gene encoding for roundabout homolog 1
*GRIK1*: gene encoding for the glutamate ionotropic receptor kainate type subunit 1
SkM: skeletal muscle (internal control for Western blot data)
