# Supplementary material for: Pharmacogenetics of response to neoadjuvant paclitaxel treatment for locally advanced breast cancer
Source: Oncotarget. 2017 Nov 15;8(63):106454–67. doi: 10.18632/oncotarget.22461 (PMC5739747; doi:10.18632/oncotarget.22461)
Supplement: Supplementary file 1 [file oncotarget-08-106454-s001.pdf]

Pharmacogenetics of response to neoadjuvant paclitaxel treatment for locally advanced breast cancer

SUPPLEMENTARY MATERIALS

Supplementary Table 1: Description of the sample by type of response* (*n* = 111)

Characteristic	Responders (*n* = 69)#	Non-responders (*n* = 42)#	*p*†
Age (years), mean ± SD	52.6 ± 10.0	50.5 ± 8.6	0.270
BMI (kg/m^2), mean ± SD	29.0 ± 4.6	28.3 ± 4.9	0.436
BMI, *n* (%)	14 (20.3)	12 (28.6)	0.149
[18.5 – 25)	32 (46.4)	14 (33.3)	
[25 – 30)	14 (20.3)	13 (31.0)	
[30 – 35)	9 (13.0)	2 (4.8)	
[35 – 40)	0 (0.0)	1 (2.4)	
≥ 40			
Breastfeeding, *n* (%)	50 (80.7)	27 (75.0)	0.512
Age at first birth (years), mean ± SD	22.4 ± 6.0	20.0 ± 4.4	0.109
Type 2 diabetes, *n* (%)	11 (15.9)	4 (9.5)	0.403
HTN, *n* (%)	15 (21.7)	10 (23.8)	0.800
Menarche (years), mean ± SD	12.9 ± 1.5	12.7 ± 2.0	0.606
**Premenopausal, *n* (%)**	**25 (36.2)**	**25 (59.5)**	**0.017**
Metformin use, *n* (%)	5 (9.1)	4 (11.1)	0.736
Hormonal exposure^, *n* (%)	13 (21.3)	15 (37.5)	0.076
Pathology report, *n* (%)			1.000
IDC	61 (88.4)	37 (88.1)	
ILC	6 (8.7)	3 (7.1)	
IDC/ILC	1 (1.5)	1 (2.4)	
Other	1 (1.5)	1 (2.4)	
Neoadjuvant trastuzumab, *n* (%)	10 (14.5)	3 (7.1)	0.364
TNM staging, *n* (%)			0.889
IIA	4 (5.9)	1 (2.4)	
IIB	8 (11.8)	7 (17.1)	
IIIA	33 (48.5)	20 (48.8)	
IIIB	18 (26.5)	11 (26.8)	
IIIC	5 (7.4)	2 (4.9)	
Molecular subtype, *n* (%)			0.505
Luminal A	29 (47.5)	24 (61.5)	
Luminal B	10 (16.4)	6 (15.4)	
Her2-enriched	10 (16.4)	3 (7.7)	
Triple-negative	12 (19.7)	6 (15.4)	
Tumor grade, *n* (%)			**0.005**
Well-differentiated	**3 (7.1)**	**7 (24.1)**	
Undifferentiated	**19 (45.2)**	**14 (48.3)**	
Poorly-differentiated	**20 (47.6)**	**8 (27.6)**	

*Numbers may not sum to totals due to missing data, and column percentages may not sum to 100% due to rounding.

^Being exposed to synthetic estrogen or estrogen-progestin oral contraceptives.

†P-value for Student's t-test (continuous variable) or χ^2 test (categorical variable) [Fisher's exact test when appropriate].

#Response to treatment was stratified based on the revised RECIST criteria. Responders were those women who had at least 30% decrease in primary tumor size, i.e. partial or complete response.

BMI: Body mass index, HTN: Hypertension.

In bold significant characteristics at the 0.05 level.

Supplementary Table 2: Minor allele frequencies stratified by response to paclitaxel (*n* = 111)

				Responders (*n* = 69)#	Non-responders (*n* = 42)#	
CHR	Gene	SNP	A1	MAF	MAF	$p^{\dagger}$
1	LPHN2	rs371363	T	0.234	0.167	0.048
3	ROBO1	rs997274	C	0.143	0.090	0.255
3	ROBO1	rs1355983	G	0.148	0.141	0.140
5	SGCD	rs7715464	A	0.258	0.218	0.340
5	SGCD	rs931798	A	0.234	0.231	0.384
5	SGCD	rs7731517	T	0.125	0.128	0.483
8	SNTG1	rs318885	T	0.000	0.038	1.000
13	DCT	rs727299	T	0.039	0.026	1.000
16	SLX4	rs714181	A	0.125	0.154	0.197
21	GRIK1	rs363599	A	0.023	0.090	0.255
21	GRIK1	rs457531	T	0.117	0.077	1.000

†*P*-value for HWE χ^2 test evaluated at the 0.01 level.
CHR – Chromosome, SNP – rs ID, A1 – least frequent allele in the sample, MAF – minor allele frequency, *p* – significance of HWE in controls.
#Response to treatment was stratified based on the revised RECIST criteria. Responders were those women who had at least 30% decrease in primary tumor size, i.e. partial or complete response.

Supplementary Table 3: Differences between tumor DNA and genomic DNA and their agreement

					Tumor DNA (*n* = 10)	Genomic DNA (*n* = 10)		
CHR	Gene	SNP	A1	A2	A1/Ht/A2	A1/Ht/A2	%a	$\varkappa_w$
1	LPHN2	rs371363	T	C	0/5/5	0/5/5	100	1.00
3	ROBO1	rs997274	C	T	1/2/7	1/2/7	100	1.00
3	ROBO1	rs1355983	G	T	3/1/6	3/1/6	100	1.00
5	SGCD	rs7715464	A	G	0/4/6	0/4/6	100	1.00
5	SGCD	rs931798	A	G	0/2/8	0/2/8	100	1.00
5	SGCD	rs7731517	T	G	0/2/8	0/2/8	100	1.00
8	SNTG1	rs318885	T	G	0/1/9	0/1/9	100	1.00
13	DCT	rs727299	T	C	0/0/10	0/0/10	100	1.00
16	SLX4	rs714181	A	G	1/3/6	1/3/6	100	1.00
21	GRIK1	rs363599	A	G	0/0/10	0/0/10	100	1.00
21	GRIK1	rs457531	T	C	0/0/8†	0/0/10	100	1.00

CHR: Chromosome, SNP: rs ID, A1: least frequent allele in the sample, A2: most frequent allele in the sample, %a: percent in agreement between genomic and tumor DNA regarding genotype information, $\varkappa$: Weighted Kappa statistic for agreement between genomic and tumor DNA regarding genotype information.
† In two cases, the tumor DNA was suboptimal to get a precise reading.

Supplementary Table 4: Unadjusted associations between alleles and change tumor size (Δ%) after paclitaxel (*n* = 111)

Gene	SNP	Alleles*	β (95% CI)	$p^{\dagger}$	Adjusted R^2
LPHN2	rs371363	**CC**	**–0.248 (–0.353, –0.144)**	<**0.0001**	**0.140**
		**CT**	**–0.261 (–0.433, –0.089)**	**0.003**	
		**TT**	**0.662 (0.171, 1.154)**	**0.009**	
ROBO1	rs997274	**TT**	**–0.298 (–0.397, –0.200)**	**<0.0001**	0.014
		CT	–0.020 (–0.287, 0.247)	NS	
		CC	–0.355 (–0.733, 0.023)	NS	
ROBO1	rs1355983	**TT**	**–0.314 (–0.416, –0.212)**	**<0.0001**	0.001
		TG	–0.033 (–0.269, 0.204)	NS	
		GG	–0.056 (–0.439, 0.327)	NS	
SGCD	rs7715464	**GG**	**–0.294 (–0.401, –0.187)**	**<0.0001**	0.007
		AG	0.020 (–0.228, 0.267)	NS	
		AA	–0.193 (–0.434, 0.049)	NS	
SGCD	rs931798	**GG**	**–0.315 (–0.422, –0.208)**	**<0.0001**	0.001
		AG	0.079 (–0.158, 0.315)	NS	
		AA	–0.148 (–0.403, 0.107)	NS	
SGCD	rs7731517	**GG**	**–0.294 (–0.395, –0.192)**	**<0.0001**	0.014
		GT	–0.128 (–0.344, 0.089)	NS	
		TT	–0.099 (–0.742, 0.546)	NS	
SNTG1	rs318885	**GG**	**–0.338 (–0.426, –0.250)**	**<0.0001**	0.029
		**GT**	**0.527 (0.009, 1.045)**	**0.046**	
DCT	rs727299	**CC**	**–0.319 (–0.411, –0.227)**	**<0.0001**	0.002
		CT	–0.042 (–0.457, 0.373)	NS	
		TT	–0.168 (–1.078, 0.742)	NS	
SLX4	rs714181	**GG**	**–0.286 (–0.388, –0.185)**	**<0.0001**	0.012
		AG	–0.172 (–0.380, 0.037)	NS	
		AA	0.191 (–0.448, 0.829)	NS	
GRIK1	rs363599	**GG**	**–0.355 (–0.442, –0.267)**	**<0.0001**	**0.104**
		AG	0.222 (–0.091, 0.535)	NS	
		**AA**	**1.497 (0.644, 2.351)**	**<0.0001**	
GRIK1	rs457531	**CC**	**–0.298 (–0.396 – –0.199)**	**<0.0001**	0.014
		CT	–0.138 (–0.377 – 0.101)	NS	
		TT	–0.131 (–0.775 – 0.512)	NS	

*Alleles displayed ordered from the most to the least frequent combination in our population.
†*p*-value for β significance
NS: not significant at the 0.05 level.
We took the most common allele for each case and set it as reference. Effects displayed first as those of the intercept for each model.
In bold significant predictors at the 0.05 level.
All these effects follow:

$$\Delta\%\,\text{response} = \frac{(\text{Phase}\,2_{\text{Postpaclitaxel}} - \text{Phase}\,1_{\text{Prepaclitaxel}})}{(\text{Phase}\,1_{\text{Prepaclitaxel}})} \sim \text{SNP}\begin{pmatrix} AA_{00} \\ Aa_{01} \\ aa_{10} \end{pmatrix} + \varepsilon$$

Where: AA is the most frequent allele taken as reference.

Supplementary Table 5: Unadjusted associations between clinic and demographic characteristics and change tumor size (Δ%) after paclitaxel (n = 111)

Characteristic	β (95% CI)^	$p^{\dagger}$	Adjusted R^2
Age (years)			0.002
Intercept	–0.221	NS	
Variable	–0.002	NS	
BMI (kg/m²)			0.007
Intercept	0.009	NS	
Variable	–0.012	NS	
Breastfeeding			0.002
Intercept [No]	**–0.313 (–0.449, –0.176)**	**<0.0001**	
Yes	–0.010	NS	
Type 2 diabetes			<0.0001
Intercept [No]	**–0.332 (–0.422, –0.242)**	**<0.0001**	
Yes	–0.008	NS	
HTN			0.003
Intercept [No]	**–0.347 (–0.442, –0.252)**	**<0.0001**	
Yes	0.061	NS	
Menarche (years)			0.001
Intercept	–0.415	NS	
Variable	0.006	NS	
Premenopausal			0.021
Intercept [No]	**–0.402 (–0.514, –0.291)**	**<0.0001**	
Yes	0.154	NS	
Metformin use			0.005
Intercept [No]	**–0.332 (–0.427, –0.236)**	**<0.0001**	
Yes	0.010	NS	
Hormonal exposure^			**0.082**
Intercept [No]	**–0.406 (–0.507, –0.305)**	**<0.0001**	
Yes	**0.304 (0.112, 0.496)**	**0.002**	
Pathology report			0.001
Intercept [IDC]	**–0.328 (–0.418, –0.238)**	**<0.0001**	
ILC	–0.048	NS	
IDC/ILC	–0.039	NS	
Other	–0.023	NS	
Neoadjuvant trastuzumab			0.007
Intercept [No]	**–0.320 (–0.409, –0.231)**	**<0.0001**	
Yes	–0.115	NS	
TNM staging			0.016
Intercept [IIA]	**–0.530 (–0.930, –0.130)**	**0.010**	
IIB	0.190	NS	
IIIA	0.179	NS	
IIIB	0.241	NS	
IIIC	0.308	NS	
Molecular subtype, *n* (%)			0.013
Intercept [Luminal]	**–0.305 (–0.412, –0.198)**	**<0.0001**	
Her2-enriched	–0.152	NS	
Triple-negative	0.001	NS	
Tumor grade, *n* (%)			0.014
Intercept [Well-differentiated]	–0.074	NS	
Undifferentiated			
Poorly-differentiated	–0.303	NS	
	–0.222	NS	

†*p*-value for β significance
^95% confidence interval for the estimated coefficient if significant.
BMI: Body mass index, HTN: Hypertension.
NS: not significant at the 0.05 level.
All these effects follow:

$$\Delta\%\,\text{response} = \frac{(\text{Phase}\,2_{\text{Postpaclitaxel}} - \text{Phase}\,1_{\text{Prepaclitaxel}})}{(\text{Phase}\,1_{\text{Prepaclitaxel}})} \sim \text{Feature} + \varepsilon$$

Supplementary Table 5.1: Adjusted haplotype associations with tumor response to paclitaxel for *ROBO1* gene (*n* =111)

#	rs1355983	rs997274	HF	*p*_a†	*p*_d†	*p*_r†
**1**	**T**	**C**	**0.031**	**0.038**	0.052	–
2	G	C	0.091	0.521	0.682	–
3	G	T	0.054	0.841	0.851	–
4	T	T	0.824	0.217	0.234	0.307

#Two single-nucleotide polymorphism (SNP) haplotype configuration.
†asymptotic χ^2 1 df *p*-value, calculated from the square of the score statistic per mode of inheritance (a = additive, d = dominant, *r* = recessive).
HF: Haplotype frequency.
– Not able to calculate.
In bold significant haplotypes at the 0.05 level.

Supplementary Table 5.2: Multivariable linear regression model of tumor response to paclitaxel adjusting for *ROBO1* gene haplotypes

#	rs1355983	rs1355983	HF	Adjusted β (95% CI)*	*p*†
1	G	C	0.084	–1.00 (–27.23, 25.24)	0.941
2	G	T	0.056	–12.71 (–41.32, 15.89)	0.387
**3**	**T**	**C**	**0.026**	**–45.40 (–92.97, 2.17)**	**0.066**
4	T	T	0.834	Reference	–

#Two single-nucleotide polymorphism (SNP) haplotype configuration.
*Effects adjusted for ever being exposed to estrogen, clinical stage, molecular subtype, and hormonal status.
†*p*-value for adjusted β significance
HF: Haplotype frequency.
In bold marginally significant haplotypes at the 0.05 level.

Supplementary Table 5.3: Adjusted haplotype associations with tumor response to paclitaxel for *SGCD* gene (*n* =111)

#	rs7715464	rs931798	rs7731517	HF	*p*_a†	*p*_d†	*p*_r†
1	A	A	T	0.074	0.281	0.347	–
2	A	A	G	0.139	0.571	0.576	0.690
3	G	G	T	0.042	0.926	0.925	–
4	G	G	G	0.695	0.270	0.166	0.510

#Three single-nucleotide polymorphism (SNP) haplotype configuration.
†asymptotic χ^2 1 df *p*-value, calculated from the square of the score statistic per mode of inheritance (a = additive, d = dominant, *r* = recessive).
HF: Haplotype frequency.
– Not able to calculate.

Supplementary Table 5.4: Multivariable linear regression model of tumor response to paclitaxel adjusting for *SGCD* gene haplotypes

#	rs7715464	rs931798	rs7731517	HF	Adjusted β (95% CI)*	$p^{\dagger}$
1	A	A	G	0.139	–6.52 (–26.24, 13.21)	0.520
2	A	A	T	0.080	–1.25 (–30.66, 28.15)	0.934
3	A	G	G	0.012	–9.83 (–61.31, 41.66)	0.710
4	G	A	G	0.025	8.60 (–30.63, 47.83)	0.669
5	G	G	T	0.054	–7.54 (–44.32, 29.23)	0.689
6	G	G	G	0.690	Reference	–

#Three single-nucleotide polymorphism (SNP) haplotype configuration.
*Effects adjusted for ever being exposed to estrogen, clinical stage, molecular subtype, and hormonal status.
†*p*-value for adjusted β significance
HF: Haplotype frequency.

Supplementary Table 6: Median AU between breast tumors and healthy controls (Mann-Whitney U results by hypotheses tests).

Gene product	Breast control (AU) Median (IQR)	Breast tumor (AU) Median (IQR)	Ratio	One-sided ($p > Z$)	Two-sided ($p > \|Z\|$)
Delta sarcoglycan	1.200 (0.212)	0.827 (0.124)	0.689	0.0368	0.0736
Syntrophin gamma 1	0.767 (0.088)	0.445 (0.195)	0.580	0.0368	0.0736
LPHN2	1.399 (0.222)	0.773 (0.286)	0.553	0.0184	0.0369
ROBO1	1.035 (0.106)	0.790 (0.313)	0.763	0.0184	0.0369
GRIK1	1.098 (0.050)	0.627 (0.173)	0.571	0.0184	0.0369

AU: Arbitrary units, intensities normalized to β-tubulin, IQR: Interquartile Range.
